# Neuroinflammatory Remodeling by Type 2 Immune Pathways Links Allergic Signaling to Neurodegenerative Disease

**DOI:** 10.3390/cells15110984

**Published:** 2026-05-27

**Authors:** Orion N. Schuldt, Sydney R. Leitch, Lauren K. Jones, Porter R. Buckley, Brad E. Morrison

**Affiliations:** Department of Biological Sciences, Boise State University, Boise, ID 83725, USA

**Keywords:** allergic inflammation, type 2 immunity, IL-4/IL-13 signaling, histamine, neurodegeneration

## Abstract

**Highlights:**

**What are the main findings?**

- Allergic/type 2 immunity regulators, such as IL-4/IL-13 pathways and histamine, are capable of modifying the neuroinflammatory milieu and influencing neuronal and glial function in various neurodegenerative conditions.
- Among the diseases reviewed, Parkinson’s disease has the closest relationship with the mechanism involving allergic-type immune signaling pathways, while Alzheimer’s disease shows better epidemiologic proof and mechanism-based evidence based on certain conditions.

**What are the implications of the main findings?**

- This provides support for a new model where immunological signaling related to allergies may affect the pathophysiology of neurodegeneration in disease-specific ways, instead of being a universal contributor.
- Stratified mechanistic approaches are necessary to determine whether Type 2 pathways are being used for biomarker, disease-modifying, or therapeutic purposes. These investigations will need to differentiate between peripheral allergy-driven immune processes versus brain-resident signaling, and must also take into consideration sex differences, pharmacologic treatment, disease stage, and type of neuropathology.

**Abstract:**

The hallmarks of allergic diseases are Type 2 immunity, including IL-4 and IL-13 production, IgE antibody generation, mast cell and basophil activation, histamine release, and eosinophil activation. There are many routes by which such mediators can influence CNS biology, including cytokine entry or signaling via brain barrier receptors; leukocyte trafficking across activated barriers; cytokine signaling via circumventricular organ sites or dural immune compartments; vagus nerve afferent signaling; mast cell degranulation; and histamine neuromodulation. Neuroinflammation is a common hallmark of many neurodegenerative diseases, but whether and to what degree allergic/type 2 immune biology may be involved depends on the specific disease stage and pathology. Here, we assess studies connecting the roles of IL-4/IL-13 signaling, IgE/mast cell activation, eosinophil-attractive chemokines, and histamines in Parkinson’s disease, Alzheimer’s disease, multiple sclerosis, amyotrophic lateral sclerosis, dementia with Lewy bodies, Huntington’s disease, prion disease, and tauopathy/atypical parkinsonism. Mechanisms appear most clear in the case of Parkinson’s disease, where epidemiology suggests an important role in dementia/Alzheimer’s disease, while for other neurodegenerative conditions the evidence is less compelling and may be either mechanistic or modulatory. Confounding issues include sex differences, drug exposures, comorbid conditions, socioeconomic factors, and coexisting inflammatory diseases. Finally, we suggest a strategy based on longitudinal immune phenotyping, CNS biomarkers, and pathway manipulation to assess the relationship between allergic immune signaling and neurodegeneration.

## 1. Introduction

Allergies and inflammatory conditions that involve them are common in clinical practice and have various clinical manifestations. Allergy is an effect of a type 2 immune response. Generally, a type 2 response involves IL-4 and IL-13, IgE, mast cell and basophil activation, histamine release, and eosinophil activation through chemokines. The type 2 axis of immunity is traditionally associated with asthma, allergic rhinitis, atopic dermatitis, food allergy, and anaphylaxis.

The group of neurodegenerative diseases represents a significant and ever-growing threat for public health in terms of burden and incidence. Such diseases as Alzheimer’s disease (AD) and Parkinson’s disease (PD) feature protein deposition, neuronal loss, synapse malfunction, and chronic inflammation of the Central Nervous System (CNS). While different in initial injury and clinical picture, the inflammatory milieu of these diseases may result, at least in part, from the peripheral immune response and immune signaling at CNS interfaces.

Peripheral allergic inflammation may signal to the brain through different non-exclusive ways. Cytokines circulating in the bloodstream can act at the level of the BBB by engaging receptors on the surface of endothelium and perivascular cells, can be translocated via specific barriers, or may cause the production of secondary mediators in the neurovascular unit. Inflammation may alter the BBB through affecting tight junctions and permeability, thus allowing the trafficking of leukocytes via adhesion molecules and chemokines. Moreover, circumventricular organs are areas where bloodborne inflammatory signals can reach the brain easier, while the vagus nerve pathway allows sending inflammatory information from the periphery to the brainstem without entering the brain parenchyma itself [1,2,3].

The interface that links IgE biology with neurodegeneration may be another mechanism of cross-talk between allergic and neurological processes. Mast cells are effector cells in allergic reaction triggered by IgE; however, mast cells also populate areas near the brain, including the meninges and dura mater. Degranulation of these cells can produce a number of agents such as histamine, proteases, cytokines, and lipids that can affect vascular tone, permeability of BBB, glia activation, fluid flow in cerebrospinal liquid, and neuroimmune signaling [4,5,6]. As a result, the effects of IgE should not only be considered from the standpoint of epidemiology and risk factors but also as a possible means through which allergic inflammation may modulate CNS immunity.

Observational human studies point to associations between common allergies and the risk of neurodegenerative outcomes like dementia and AD [7,8,9]. These findings serve as suggestions rather than a proof of causality, and the issue requires careful examination to consider all the factors like age, sex, socio-economic position, environment exposure, disturbed sleep, medication, anticholinergic burden, vascular disease, obesity, smoking, and other inflammatory pathologies [10,11,12,13].

**Figure 1:**
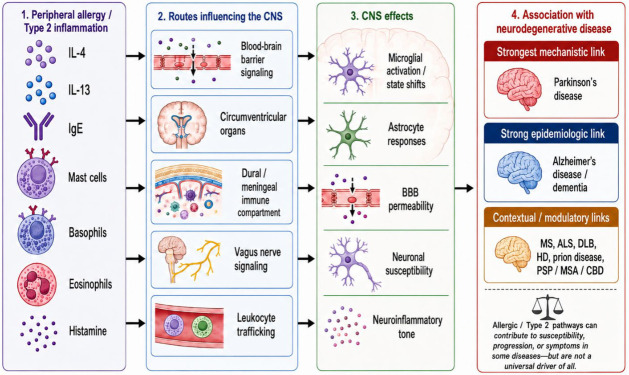
Diagram of peripheral allergy-to-brain signaling. Type 2 allergic responses have the potential to impact CNS function by way of various peripheral mediators such as IL-4, IL-13, IgE, mast cells, basophils, eosinophils, and histamine. Such signaling can reach the brain through several mechanisms, including blood–brain barrier signaling, circumventricular organs, dural/meningeal immune compartmentalization, vagal afferents, and leukocyte migration. At the level of the CNS, these signaling modalities may affect microglial activation states, astrocyte reactivity, BBB permeability, neuronal vulnerability, and neuroinflammation overall. Presently, there is the most robust mechanistic link with Parkinson’s disease, a more robust epidemiological link with Alzheimer’s disease and dementia, and more modulatory/conditional links with MS, ALS, DLB, HD, prion diseases, PSP, MSA, and CBD. In summary, type 2 allergic responses may play a role in disease pathogenesis/progression/symptomatology specifically, without being universal mediators of neurodegeneration.

This review focuses on four biological components: (1) Type 2 cytokines, mainly IL-4 and IL-13; (2) IgE, mast cells, basophils, and related effector mechanisms; (3) histamine and its receptors; and (4) glial and vascular mechanisms linking allergic inflammation to the pathology of the brain [1,2,3,4,5,6,14,15,16]. Each disease is described with respect to what is proved or only suggested, and also in terms of whether the type 2 pathway works as a primary mechanism, a modulator of inflammation, or just a biomarker (Figure 1).

## 2. Parkinson’s Disease

Parkinson’s disease (PD) is a progressive neurodegenerative disorder characterized by aggregation of α-synuclein and loss of dopaminergic neurons in the midbrain, particularly in the substantia nigra [17]. The cell mechanisms that contribute to PD include oxidative stress, mitochondrial dysfunction, and protein handling mechanisms [18]. Although PD is primarily associated with increased age, environmental and genetic factors also contribute to PD. Recent studies suggest that immune mechanisms also contribute to PD [17,19]. This review aims to discuss PD, with a particular emphasis on immune mechanisms, particularly Type 2 immune mechanisms.

The status of IL-4 and IL-13 as primary mediators of PD pathogenesis is not yet warranted. The current scientific literature suggests the opposite view, wherein the functions of these cytokines are more appropriately seen as dependent upon their modulation of inflammatory tone and cellular susceptibility. IL-4 is capable of influencing cellular activation and cytokine secretion in microglia and macrophages. For dopaminergic neurons, the role of IL13RA1 appears to be more autonomous due to its presence in dopaminergic neurons and increased susceptibility to oxidative stress [11,14]. Additionally, IL13RA1 is a classical allergy-related receptor, essential for airway hyper-reactivity, mucus hypersecretion, and eotaxin expression. This receptor lies in the PARK12 susceptibility locus located on the chromosome arm Xq24. Il13ra1 knockout mice demonstrated resistance to chronic peripheral administration of LPS leading to dopaminergic neuron death, indicating that IL-13/IL13RA1 pathway contributes to increased susceptibility to inflammatory or oxidative damage in susceptible dopaminergic neurons but does not constitute a mediator of PD pathogenesis per se [18,20].

Histamine, a mediator of mast cells and neuromodulator of the central nervous system, represents another allergic component of relevance to PD pathogenesis. It was found to be involved in PD pathogenesis by alteration of its receptors’ expression in PD patients [21,22,23], while elevated levels of histamine were noted in the substantia nigra and putamen of PD patients [24]. It has been found that histamine promotes microglia activation and reactive oxygen species formation through stimulation of H1R and H4R receptors, while inhibition of H4R receptors has shown a protective effect in PD experiments [25,26,27]. However, there is no sufficient data regarding the IgE-dependent contribution of mast-cell degranulation into activation of histamine-related processes associated with PD [4,5,6,14].

## 3. Alzheimer’s Disease

Alzheimer’s disease, one of the most common underlying causes of dementia, is marked by the progressive deterioration of memory and cognitive functions. In addition, it is accompanied by behavioral changes. AD is marked by the accumulation of Amyloid-Beta and Tau in the brain. The role of inflammation and the immune system in AD is becoming increasingly recognized and indicates the importance of these factors in the progression of the disease. Microglia are the resident immune cells in the central nervous system. Microglia activation occurs in the presence of Amyloid-Beta accumulation in the brain and thereby affects central nervous system inflammation [28]. Allergic inflammation is mediated by unique immune mechanisms that are marked by the presence of Type 2 immunity [4,15,16].

Epidemiological studies have shown a correlation between allergic disorders and dementia and Alzheimer’s disease (AD) incidence. Population-level studies showed that people diagnosed with asthma, allergic rhinitis, or atopic dermatitis had greater dementia and AD occurrence rates than controls who did not suffer from these diseases [7]. The association of IgE-mediated allergic disorders was also found to be more common in patients with AD compared to non-sufferers [8], while the incidence of AD has been demonstrated to be elevated in people with allergic rhinitis under high particulate matter conditions [9]. Although the mentioned facts provide an explanation of the underlying biological mechanism, they should be considered cautiously since various factors, including allergic diagnoses, air pollution, disrupted sleeping, drug prescription, anticholinergics, cardiometabolic disease, access to healthcare, and social-economic aspects, can correlate with dementia status [7,8,9,10,11,12].

In turn, the impact of IL-4 and IL-13 on amyloid deposits seems to depend on the model, stage of illness, dosages, and specific cells. Administration of IL-4 or IL-13 has been associated with the decrease in amyloid-beta accumulation and enhanced cognitive performance in some AD models [29]. On the other hand, increased production of IL-4 in hippocampus has been demonstrated to cause an increase in amyloid-beta formation [30]. Thus, contradictory results may be explained by the assumption that transitory or properly localized expression of Type 2 cytokines may lead to facilitation of anti-inflammatory processes or enhancement of phagocytic/glial cell phenotypes, while long-term, excessive or improperly placed signaling may affect glial cells, interfere with amyloid processing, or provoke pathological changes. Hence, Type 2 cytokines may exhibit different influences on various brain cells (microglia, astrocytes, vascular endothelium, peripheral myeloid cells, and neurons) [29,30,31].

Summing up, it is possible to state that there is sufficient evidence for a biologically plausible link between allergic disorders and AD, but the relationship lacks evidence regarding one-way causality. The possible mechanism can be explained by the presence of IgE-mediated allergy or mast cells because their activity can influence the function of blood–brain barrier, glial cells and brain borders [4,5,6,8]. The effects of histamine receptors and antihistamines are important in regard to the discussion on AD because the effects of antihistamine medications in epidemiological research vary in accordance with the class of drugs used and cumulative exposure period [10,11,12]. Therefore, it is correct to say that AD can be considered as a condition that can be affected by allergic-type 2 pathways in particular subpopulations.

## 4. Multiple Sclerosis

MS is a chronic inflammatory demyelinating disease affecting the CNS. It is not related to other neurodegenerative conditions like Alzheimer’s disease, Parkinson’s disease, or Amyotrophic Lateral Sclerosis (ALS), nor does it fit within the category of diseases caused by a Type 2 immune response imbalance. This condition is used as a comparative tool for studying how allergenic factors might affect CNS inflammation, BBB function, and the migration of immune cells without being a primary cause of illness. The development of MS has been shown to involve the Th1/Th17 pathway, infiltration of leukocytes into the CNS, damage to oligodendrocytes, and demyelination [32].

Existing research on biomarkers does not yield a consistent pattern for MS, although overall it points towards a pattern of histamine-related signaling pathways. One such study showed that histamine and diamine oxidase levels were lower in relapsing-remitting multiple sclerosis patients compared to healthy controls [33], whereas another study showed that histamine and methylhistamine levels were not elevated in multiple sclerosis patients, but a protease associated with mast cells, namely tryptase, was significantly elevated in the cerebrospinal fluid of multiple sclerosis patients [34]. Overall, these studies are consistent with the conclusion that mast cell and histamine signaling may play a role in the pathology of multiple sclerosis [33,34].

Consequently, the Type 2 signals involved in MS should be regarded as secondary or conditional modulators. Histamine affects different receptors in a variety of ways: histamine acting on H1 and H2 receptors has been shown to play a detrimental role in EAE. However, the function of histamine on H3 and H4 receptors depends on cellular context [35]. There is no universal allergic pattern for biomarkers; however, changes in histamine turnover and increased CSF tryptase activity suggest that mast cell and histamine-related processes are implicated in certain aspects of MS [33,34]. Strategies targeting IL-4 could mitigate EAE progression in certain models, yet these studies show immunomodulation rather than a causative role for the Type 2 response in MS [36,37,38].

## 5. Amyotrophic Lateral Sclerosis

Amyotrophic lateral sclerosis (ALS) is characterized by the progressive degeneration of upper and lower motor neurons. Neuroinflammatory processes have been documented in ALS and include microglial and astrocyte activation and corresponding peripheral immune reactions. The role of inflammation in ALS is complex and multifaceted, with early stages of inflammation having neuroprotective effects and repairing tissues, and later stages of inflammation being correlated with greater neuronal damage and progression of the disease [39]. The multifaceted role of inflammation in ALS makes it highly pertinent to the current review and the potential role of cytokines associated with allergic inflammation in motor neuron survival.

New evidence suggests that cytokines related to Type 2 reactions, such as IL-4 and IL-13, may be related to the processes of ALS. Although these cytokines are related to the process of allergic reactions, it is also suggested that these cytokines may be related to the modulation of the states of glial cells and inflammation in general. The role of Type 2 reactions in ALS has also been related in other studies, suggesting that IL-4 and IL-13 may be related to the process of inflammation in ALS in a less detrimental way [40,41].

Another potential pathway for this relationship between allergic cytokines and ALS involves the action of histamine. In the setting of allergic reactions, histamine is a key mediator released from mast cells and basophils in reaction to allergens. It also has a direct effect on motor neurons and may influence survival pathways. The role of histamine in the setting of ALS was studied, and it was shown that this compound can protect motor neurons through the activation of the AKT and ERK1/2 pathways and by increasing mitochondrial function and ATP production [42]. As mitochondrial dysfunction and ATP management are key aspects of ALS, this pathway may be a common pathway that relates to allergic cytokines and ALS.

Type 2-related cytokines may or may not be involved in ALS, depending on the study or the specific stages of ALS. The general theme of the current manuscript is that cytokines, such as IL-4 and IL-13, that are part of the immune response may not necessarily play a specific role in ALS but may, depending on the situation, influence the course of the disease by modulating microglia, other immune cells, or even neurons. In fact, ALS studies from humans have shown that there are alterations in T-cell-related cytokine responses, including IL-13-related pathways [43]. As a summary, the current evidence supports that IL-4, IL-13, and histamine are potential modifiers of the neuroinflammatory tone and motor neuron survival in ALS but are not central players.

## 6. Dementia with Lewy Bodies (DLB) and Related Lewy Body Diseases

DLB is a common form of dementia caused by α-synuclein pathology and cognitive dysfunction, fluctuating cognitive function, visual hallucinations, and parkinsonism. While there are clear differences between the neurotransmitter and receptor biology of DLB and AD, the involvement of histamine is still relevant due to its association with allergies, a central nervous system (CNS) modulator for arousal/cognition, and a neuroinflammatory regulator. Binding studies show that histamine H3 receptors in the brain of DLB patients appear unaffected by disease and correlate with the presence of psychosis and hallucinations [44]. These studies provide evidence for a connection between histamine and Lewy body dementia but are insufficient to demonstrate that allergic diseases contribute to DLB.

IL-4 and IL-13 do not have as much supporting evidence in DLB compared to histamine. IL-4 is known to be upregulated in peripheral blood samples in the early stages of Lewy body disease, such as mild cognitive impairment with Lewy bodies, and does not differentiate DLB from healthy controls [45,46]. Low IL-4 was shown to correlate with faster disease progression in longitudinal analyses of DLB [46]. Similarly, IL-13 data is conflicting; while one study did not find any significant changes in cytokine expression in DLB, another study found elevated IL-13 in association with IL-10, IL-1α, and amyloid-β pathology in DLB brain tissue [47]. Overall, these results suggest that Type 2-related cytokines might play a role in the prodromal period, disease heterogeneity, or a mixture of Lewy body/amyloid pathology rather than being involved in the development of DLB [45,46,47]. Anti-histamine and dementia pharmacoepidemiology studies are relevant to the discussion of histamine but should not be used as evidence for allergic diseases and DLB [10,11,12].

## 7. Huntington’s Disease

Mutant Huntingtin causes Huntington’s disease (HD), resulting in progressive motor, cognitive, and psychiatric manifestations. The immune response has been implicated in HD, but there is limited data specifically supporting Type 2 immunity. Thus, HD should be considered a disease where Type 2 immunity biomarkers might indicate disease activity or immune system remodeling rather than an illness with well-established allergic/type 2 immune causation [48,49].

The most convincing Type 2-related findings in HD come from studies demonstrating elevated levels of IL-4 and allergy-related chemokines, such as eotaxin and eotaxin-3, in peripheral blood with some parameters associated with disease progression [48,50]. Due to the similarity in the study origin and design, a cautious evaluation of these findings is needed. In terms of the broader immunology literature, immune dysregulation has been implicated in HD, particularly in terms of innate immune system and myeloid cells, including increased cytokine release from HD myeloid cells and enhanced microglial responsiveness to inflammation [49,51].

In summary, HD findings can be considered preliminary and biomarker-focused. IL-4 and eotaxins might serve as markers of immune status changes associated with HD progression, but the current literature does not demonstrate that Type 2 immunity underlies striatal neurodegeneration in this disease. Further research should consider independent replication of the described parameters, examination of compartmentalized central nervous system cytokines, and assessment of the relevance of changes in eotaxin/IL-4 after adjustment for disease severity, patient age, gender, medications, and systemic inflammation [48,49,50,51,52].

## 8. Prion Diseases

Prion diseases are rapidly progressive neurodegenerative diseases with prion protein misfolding, gliosis, and the presence of a strong innate immune response in the central nervous system. Although prion diseases are not considered to be part of the group of allergic diseases, several studies point to the fact that the cytokines which are generally related to anti-inflammatory or Type 2 immune reactions show alterations during prion disease in humans and animal models. Indeed, in the context of Creutzfeldt–Jakob disease in humans, the analysis of cerebrospinal fluid shows that the levels of IL-4 and IL-10 are substantially elevated compared to controls. This observation supports the idea that the inflammatory environment in prion disease is not exclusively related to pro-inflammatory signals. Rather, it may also comprise compensatory Th2-related signals in the CNS during prion disease [53]. This observation is in line with the idea that the cells involved in the inflammation in prion disease, such as microglia and astrocytes, can assume different functional states during the course of the disease, including those related to the production of anti-inflammatory cytokines and tissue repair, as opposed to destructive functions [54,55].

This experimental evidence also supports a more selective role for these cytokines, as opposed to a universal effect on all Type 2-associated pathways. In murine models of prion infection, loss of IL-10 results in accelerated disease onset and progression, suggesting that IL-10 has a protective or modifying effect on prion disease pathogenesis. In contrast, loss of IL-4 or IL-13 alone does not appear to have such an accelerating effect, suggesting that these cytokines are not functionally equivalent, even though they are often considered to be part of a cytokine family with anti-inflammatory properties, or Th2 cytokines [56]. Further studies also suggest that IL-13 expression can be transiently upregulated early in prion infection in certain contexts, such as in CD14-deficient mice, suggesting that Type 2-associated cytokine responses, such as those involving IL-13, may be time-dependent and context-dependent, and not sustained throughout disease [57]. Thus, the current evidence does not support a simple model of allergy-like immune mechanisms driving prion disease, but does support a model where anti-inflammatory cytokines intersect with prion disease pathogenesis.

The above body of research suggests that IL-4, IL-10, and to a lesser extent IL-13, should be considered to be modifiers of the CNS immune environment in prion disease, rather than driving factors in disease pathogenesis. The presence of these cytokines in human CSF, and their effects in animal models, suggests a model of compartmentalization of immune responses, restraint of immune responses, or regulation of glial cell types in ongoing neurodegenerative processes [53,56]. This makes prion disease a model of interest to those considering the broader context of allergic or Type 2-associated biology in neurodegenerative diseases, since even in those diseases not classically considered to be associated with allergy, cytokines associated with anti-inflammatory and Th2-type immune responses appear to be involved in disease progression and immune/inflammatory responses in the injured brain [53,56,57].

## 9. Atypical Parkinsonian Disorders and Tauopathies

Atypical parkinsonism disorders or the tauopathy/synucleinopathy category include progressive supranuclear palsy (PSP), corticobasal degeneration (CBD), and multiple system atrophy (MSA). Nevertheless, specific data on Type 2 allergies for these diseases is still scarce. For PSP, postmortem research findings point to the expression of cytokines and the activation of microglia in involved brain areas and thus to neuroinflammation, though they do not show the presence of characteristic Th2 cytokines [58]. Concerning CBD, there are no data pointing out the involvement of IgE, mast cells, histamine, IL-4, IL-13, or eosinophil chemokines in the disease course. Hence, CBD can be classified only as a related tauopathy.

For MSA, biochemical studies in the postmortem setting demonstrate increased histamine concentrations in Parkinson’s disease but not MSA, indicating the existence of differences in the role of the histaminergic pathway between various parkinsonian disorders [24]. Therefore, the most reasonable statement regarding this category of disorders would be the following: both PSP and MSA have a disease-specific inflammatory/histaminergic profile, while CBD lacks any convincing evidence of the disease association.

## 10. Discussion

Current conceptualizations of neurodegenerative diseases emphasize the role of cellular stress responses coupled with remodeling of the immune microenvironment. Consistent with this framework, this review provides evidence that allergic/type 2 immune signaling, including IL-4/IL-13 signaling, histamine, IgE/mast-cell effectors, and eosinophil-derived chemokines, can impact neuroinflammatory tone, neuron sensitivity, glia phenotypes, blood–brain barrier integrity, and symptoms in neurodegeneration. This evidence does not confirm an all-encompassing hypothesis of Type 2 immunity driving neurodegeneration. On the contrary, the extent and polarity of effect vary between different diseases, stages, cell types, anatomic compartments, and comorbidities (Table 1) [1,2,3,5,6,14,59,60].

The following avenues should be explored in future studies to advance our understanding of allergic mechanisms in neurodegeneration. First, longitudinal human cohorts should include characterization of allergic phenotype along with immune profiling of IL-4/IL-13 signaling activity, IgE, eosinophil-related chemokines, mast-cell markers, histamine metabolites, and antihistamine usage. Second, the concept of neurodegeneration should be complemented with CSF biomarker assessment, neuroimaging indicators of neuroinflammation or blood–brain barrier abnormalities, subtype-specific neuropathology data, and disease-relevant clinical outcomes. Third, the analysis should be stratified by sex, age, stage of the disease, sleep disturbance, smoking, obesity, vascular disease, social deprivation, and pharmacotherapeutic intervention, in particular, by first- or second-generation antihistamines [10,11,12,13]. Fourth, preclinical studies need to decouple peripherally driven allergic inflammation and CNS-resident Type 2 signaling through cell-specific manipulation of IL4Rα/IL13RA1, histamine receptors, mast-cell activity, and eosinophil chemoattractants. Finally, pathway-selective manipulations should be used as causal mechanisms rather than therapeutic candidates since the same mechanism can have a protective role in one setting and pathogenic role in another.

Overall, current research provides a basis for a refined model of neurodegeneration involving allergic/type 2 immune responses as a component of more general neuroimmune milieu of neurodegeneration. These results open ample opportunities for mechanistic investigation but are far from providing unequivocal evidence of causality. Parkinson’s disease provides a most compelling case based on molecular targets of allergic-type 2 responses such as IL13RA1 and histamine biology. Conversely, Alzheimer’s disease demonstrates better epidemiological support but ambiguous mechanistic direction. In addition, multiple neurological disorders, e.g., MS, ALS, DLB, HD, prion diseases, PSP, CBD, and MSA, should be viewed as distinct biological contexts in which type 2-related mediators can shape neurodegeneration. Further studies are needed to understand the roles of these pathways as either correlative markers, modulators of the disease course, or therapeutic targets.

## Figures and Tables

**Table 1:** Evidence for the allergic/type 2 immune response pathway in neurodegenerative and neuroinflammatory disorders.

Disease	Pathway and Evidence	Direction/Strength	Interpretation
Parkinson’s disease	IL-4/IL-13, IL13RA1, histamine; human and animal studies	Predominantly negative/contextual; Moderate	Strongest link between allergy and neurodegeneration
Alzheimer’s disease	IL-4/IL-13, IgE/mast cells, histamine, allergy epidemiology; animal and pharmacoepidemiology data	Mixed/contextual; Moderate-Strong	Epidemiological support; mixed mechanisms
Multiple sclerosis	Histamine, tryptase, IL-4; human biomarker and EAE models	Secondary/contextual; Moderate	Important mediators, though MS is not a Type 2 disease
ALS	IL-4/IL-13, histamine; mechanistic and limited human studies	Limited/mixed; Limited	Type 2 modifiers, not drivers
DLB	Histamine, limited IL-4/IL-13; human pathology/biomarkers	Limited/mixed; Limited	Better histamine evidence than cytokine
Huntington’s disease	IL-4 and eotaxins; human biomarkers and broader innate immunity	Preliminary/mixed; Limited	Preliminary conclusions about Type 2
Prion diseases	IL-4, IL-10, IL-13; human CSF and animal studies	Mixed/contextual; Limited to Moderate	Cytokines affect the immune microenvironment rather than drive disease processes
PSP, MSA, CBD insufficient	MSA histamine; PSP cytokine microenvironment; no direct CBD Type 2 link found	Limited/unclear; Limited	PSP and MSA show inflammatory differences; CBD explicitly qualified

“Directionality” describes the nature of the effect on disease biology as being negative, positive, uncertain, conditional, secondary/regulatory, weak, or unknown. “Strength of evidence” considers the number of studies, degree of independence, disease relevance, direct involvement of the brain, and level of mechanistic understanding. Weak evidence is defined by few studies, an indirect relationship, a lack of independent groups, and nonspecificity to the disease under study. Moderately strong evidence is provided by several independent studies in humans or animals with potential biological plausibility. Strong evidence includes human epidemiology or mechanistic findings but is not enough to prove causality. Conflict is based on evidence from a single study or conflicting results and was scored more critically than disease-specific human data and direct CNS evidence.

## Data Availability

No new data were created or analyzed in this study.

## References

[1] Banks W. (2005). Blood-Brain Barrier Transport of Cytokines: A Mechanism for Neuropathology. Curr. Pharm. Des..

[2] Erickson M.A., Banks W.A. (2018). Neuroimmune Axes of the Blood–Brain Barriers and Blood–Brain Interfaces: Bases for Physiological Regulation, Disease States, and Pharmacological Interventions. Pharmacol. Rev..

[3] Takeshita Y., Ransohoff R.M. (2012). Inflammatory cell trafficking across the blood–brain barrier: Chemokine regulation and in vitro models. Immunol. Rev..

[4] Galli S.J., Tsai M. (2012). IgE and mast cells in allergic disease. Nat. Med..

[5] Mamuladze T., Kipnis J. (2023). Type 2 immunity in the brain and brain borders. Cell. Mol. Immunol..

[6] Mamuladze T., Zaninelli T.H., Smyth L.C., Wu Y., Abramishvili D., Silva R., Imbiakha B., Verhaege D., Du S., Papadopoulos Z. (2025). Mast cells regulate the brain-dura interface and CSF dynamics. Cell.

[7] Joh H., Kwon H., Son K.Y., Yun J.M., Cho S.H., Han K., Park J., Cho B. (2022). Allergic Diseases and Risk of Incident Dementia and Alzheimer’s Disease. Ann. Neurol..

[8] Bozek A., Krupka-Olek M., Krupka A.K. (2024). IgE-mediated allergic diseases are associated with Alzheimer’s disease. Allergy.

[9] Li R.-L., Ho Y.-C., Luo C.-W., Lee S.-S., Kuan Y.-H. (2019). Influence of PM_2.5_ Exposure Level on the Association between Alzheimer’s Disease and Allergic Rhinitis: A National Population-Based Cohort Study. Int. J. Environ. Res. Public Health.

[10] Su C.-H., Huang K.-H., Yang Y., Gau S.-Y., Chung N.-J., Wu P.-T., Tsai T.-H., Lee C.-Y. (2024). Cumulative Dose Effects of H1 Antihistamine Use on the Risk of Dementia in Patients with Allergic Rhinitis. J. Allergy Clin. Immunol. Pract..

[11] Yang C.-C., Chien W.-C., Chung C.-H., Lai C.-Y., Tzeng N.-S. (2022). The Usage of Histamine Type 1 Receptor Antagonist and Risk of Dementia in the Elderly: A Nationwide Cohort Study. Front. Aging Neurosci..

[12] Andersson N.W., Elberling J., Hviid A. (2025). Second-generation antihistamine use and risk of dementia: Nationwide cohort study. J. Allergy Clin. Immunol. Pract..

[13] Lopez-Lee C., Kodama L., Gan L. (2022). Sex Differences in Neurodegeneration: The Role of the Immune System in Humans. Biol. Psychiatry.

[14] Thangam E.B., Jemima E.A., Singh H., Baig M.S., Khan M., Mathias C.B., Church M.K., Saluja R. (2018). The Role of Histamine and Histamine Receptors in Mast Cell-Mediated Allergy and Inflammation: The Hunt for New Therapeutic Targets. Front. Immunol..

[15] Zhu J. (2015). T helper 2 (Th2) cell differentiation, type 2 innate lymphoid cell (ILC2) development and regulation of interleukin-4 (IL-4) and IL-13 production. Cytokine.

[16] Bao K., Reinhardt R.L. (2015). The differential expression of IL-4 and IL-13 and its impact on type-2 immunity. Cytokine.

[17] Marogianni C., Sokratous M., Dardiotis E., Hadjigeorgiou G.M., Bogdanos D., Xiromerisiou G. (2020). Neurodegeneration and Inflammation—An Interesting Interplay in Parkinson’s Disease. Int. J. Mol. Sci..

[18] Morrison B.E., Marcondes M.C.G., Nomura D.K., Sanchez-Alavez M., Sanchez-Gonzalez A., Saar I., Kim K.-S., Bartfai T., Maher P., Sugama S. (2012). Cutting Edge: IL-13Rα1 Expression in Dopaminergic Neurons Contributes to Their Oxidative Stress-Mediated Loss Following Chronic Peripheral Treatment with Lipopolysaccharide. J. Immunol..

[19] Chelpin M.E., Vorup-Jensen T. (2017). Targets and Mechanisms in Prevention of Parkinson’s Disease through Immunomodulatory Treatments. Scand. J. Immunol..

[20] Bok E., Cho E.J., Chung E.S., Shin W.-H., Jin B.K. (2018). Interleukin-4 Contributes to Degeneration of Dopamine Neurons in the Lipopolysaccharide-treated Substantia Nigra in vivo. Exp. Neurobiol..

[21] Anichtchik O.V., Peitsaro N., Rinne J.O., Kalimo H., Panula P. (2001). Distribution and Modulation of Histamine H3 Receptors in Basal Ganglia and Frontal Cortex of Healthy Controls and Patients with Parkinson’s Disease. Neurobiol. Dis..

[22] Anichtchik O.V., Huotari M., Peitsaro N., Haycock J.W., Männistö P.T., Panula P. (2000). Modulation of histamine H_3_ receptors in the brain of 6-hydroxydopamine-lesioned rats. Eur. J. Neurosci..

[23] Sharma A., Muresanu D.F., Patnaik R., Menon P.K., Tian Z.R., Sahib S., Castellani R.J., Nozari A., Lafuente J.V., Buzoianu A.D. (2021). Histamine H3 and H4 Receptors Modulate Parkinson’s Disease Induced Brain Pathology. Neuroprotective Effects of Nanowired BF-2649 and Clobenpropit with Anti-Histamine-Antibody Therapy. Progress in Brain Research.

[24] Rinne J.O., Anichtchik O.V., Eriksson K.S., Kaslin J., Tuomisto L., Kalimo H., Röyttä M., Panula P. (2002). Increased brain histamine levels in Parkinson’s disease but not in multiple system atrophy. J. Neurochem..

[25] Rocha S.M., Saraiva T., Cristóvão A.C., Ferreira R., Santos T., Esteves M., Saraiva C., Je G., Cortes L., Valero J. (2016). Histamine induces microglia activation and dopaminergic neuronal toxicity via H1 receptor activation. J. Neuroinflamm..

[26] Fang Q., Xicoy H., Shen J., Luchetti S., Dai D., Zhou P., Qi X.-R., Martens G.J., Huitinga I., Swaab D.F. (2021). Histamine-4 receptor antagonist ameliorates Parkinson-like pathology in the striatum. Brain Behav. Immun..

[27] Zhou P., Homberg J.R., Fang Q., Wang J., Li W., Meng X., Shen J., Luan Y., Liao P., Swaab D.F. (2019). Histamine-4 receptor antagonist JNJ7777120 inhibits pro-inflammatory microglia and prevents the progression of Parkinson-like pathology and behaviour in a rat model. Brain Behav. Immun..

[28] Cameron B., Landreth G.E. (2010). Inflammation, microglia, and Alzheimer’s disease. Neurobiol. Dis..

[29] Lyons A., Griffin R.J., E Costelloe C., Clarke R.M., A Lynch M. (2007). IL-4 attenuates the neuroinflammation induced by amyloid-β in vivo and in vitro. J. Neurochem..

[30] Latta C.H., Sudduth T.L., Weekman E.M., Brothers H.M., Abner E.L., Popa G.J., Mendenhall M.D., Gonzalez-Oregon F., Braun K., Wilcock D.M. (2015). Determining the role of IL-4 induced neuroinflammation in microglial activity and amyloid-β using BV2 microglial cells and APP/PS1 transgenic mice. J. Neuroinflamm..

[31] Szczepanik A. (2001). IL-4, IL-10 and IL-13 modulate Aβ(1–42)-induced cytokine and chemokine production in primary murine microglia and a human monocyte cell line. J. Neuroimmunol..

[32] Dobson R., Giovannoni G. (2018). Multiple sclerosis—A review. Eur. J. Neurol..

[33] Zadeh A.R., Falahatian M., Alsahebfosoul F. (2018). Serum levels of histamine and diamine oxidase in multiple sclerosis. Am. J. Clin. Exp. Immunol..

[34] Rozniecki J.J., Hauser S.L., Stein M., Lincoln R., Theoharides T.C. (1995). Elevated mast cell tryptase in cerebrospinal fluid of multiple sclerosis patients. Ann. Neurol..

[35] Saligrama N., Noubade R., Case L.K., del Rio R., Teuscher C. (2012). Combinatorial roles for histamine H_1_-H_2_ and H_3_-H_4_ receptors in autoimmune inflammatory disease of the central nervous system. Eur. J. Immunol..

[36] Furlan R., Poliani P., Marconi P., Bergami A., Ruffini F., Adorini L., Glorioso J., Comi G., Martino G. (2001). Central nervous system gene therapy with interleukin-4 inhibits progression of ongoing relapsing–remitting autoimmune encephalomyelitis in Biozzi AB/H mice. Gene Ther..

[37] Vogelaar C.F., Mandal S., Lerch S., Birkner K., Birkenstock J., Bühler U., Schnatz A., Raine C.S., Bittner S., Vogt J. (2018). Fast direct neuronal signaling via the IL-4 receptor as therapeutic target in neuroinflammation. Sci. Transl. Med..

[38] Monteiro L., Souza-Machado A., Menezes C., Melo A. (2010). Association between allergies and multiple sclerosis: A systematic review and meta-analysis. Acta Neurol. Scand..

[39] McCombe P.A., Henderson R.D. (2011). The Role of Immune and Inflammatory Mechanisms in ALS. Curr. Mol. Med..

[40] Stacchiotti C., di Regnella S.M., Cinotti M., Spalloni A., Volpe E. (2025). Neuroinflammation and Amyotrophic Lateral Sclerosis: Recent Advances in Anti-Inflammatory Cytokines as Therapeutic Strategies. Int. J. Mol. Sci..

[41] Rossi C., Cusimano M., Zambito M., Finardi A., Capotondo A., Garcia-Manteiga J.M., Comi G., Furlan R., Martino G., Muzio L. (2018). Interleukin 4 modulates microglia homeostasis and attenuates the early slowly progressive phase of amyotrophic lateral sclerosis. Cell Death Dis..

[42] Volonté C., Apolloni S., Sabatelli M. (2019). Histamine beyond its effects on allergy: Potential therapeutic benefits for the treatment of Amyotrophic Lateral Sclerosis (ALS). Pharmacol. Ther..

[43] Shi N., Kawano Y., Tateishi T., Kikuchi H., Osoegawa M., Ohyagi Y., Kira J.-I. (2007). Increased IL-13-producing T cells in ALS: Positive correlations with disease severity and progression rate. J. Neuroimmunol..

[44] Lethbridge N.L., Chazot P.L. (2016). Ligand autoradiographical quantification of histamine H 3 receptor in human dementia with Lewy bodies. Pharmacol. Res..

[45] King E., O’brien J.T., Donaghy P., Morris C., Barnett N., Olsen K., Martin-Ruiz C., Taylor J.-P., Thomas A.J. (2017). Peripheral inflammation in prodromal Alzheimer’s and Lewy body dementias. J. Neurol. Neurosurg. Psychiatry.

[46] Thomas A.J., Hamilton C.A., Donaghy P.C., Martin-Ruiz C., Morris C.M., Barnett N., Olsen K., Taylor J., O’BRien J.T. (2020). Prospective longitudinal evaluation of cytokines in mild cognitive impairment due to AD and Lewy body disease. Int. J. Geriatr. Psychiatry.

[47] Chai Y.L., Lee J.H., Chong J.R., Ballard C., Francis P.T., Kennedy B.K., Arumugam T.V., Chen C.P., Aarsland D., Lai M.K.P. (2023). Inflammatory panel cytokines are elevated in the neocortex of late-stage Alzheimer’s disease but not Lewy body dementias. J. Neuroinflamm..

[48] Björkqvist M., Wild E.J., Thiele J., Silvestroni A., Andre R., Lahiri N., Raibon E., Lee R.V., Benn C.L., Soulet D. (2008). A novel pathogenic pathway of immune activation detectable before clinical onset in Huntington’s disease. J. Exp. Med..

[49] Träger U., Andre R., Lahiri N., Magnusson-Lind A., Weiss A., Grueninger S., McKinnon C., Sirinathsinghji E., Kahlon S., Pfister E.L. (2014). HTT-lowering reverses Huntington’s disease immune dysfunction caused by NFκB pathway dysregulation. Brain.

[50] Wild E., Magnusson A., Lahiri N., Krus U., Orth M., Tabrizi S.J., Björkqvist M. (2011). Abnormal peripheral chemokine profile in Huntington’s disease. PLoS Curr..

[51] Connolly C., Magnusson-Lind A., Lu G., Wagner P., Southwell A., Hayden M., Björkqvist M., Leavitt B. (2016). Enhanced immune response to MMP3 stimulation in microglia expressing mutant huntingtin. Neuroscience.

[52] Huber A.K., Giles D.A., Segal B.M., Irani D.N. (2018). An emerging role for eotaxins in neurodegenerative disease. Clin. Immunol..

[53] Stoeck K., Bodemer M., Ciesielczyk B., Meissner B., Bartl M., Heinemann U., Zerr I. (2005). Interleukin 4 and Interleukin 10 Levels Are Elevated in the Cerebrospinal Fluid of Patients With Creutzfeldt-Jakob Disease. Arch. Neurol..

[54] Zhu C., Herrmann U.S., Falsig J., Abakumova I., Nuvolone M., Schwarz P., Frauenknecht K., Rushing E.J., Aguzzi A. (2016). A neuroprotective role for microglia in prion diseases. J. Exp. Med..

[55] Smith H.L., Freeman O.J., Butcher A.J., Holmqvist S., Humoud I., Schätzl T., Hughes D.T., Verity N.C., Swinden D.P., Hayes J. (2020). Astrocyte Unfolded Protein Response Induces a Specific Reactivity State that Causes Non-Cell-Autonomous Neuronal Degeneration. Neuron.

[56] Thackray A.M., McKenzie A.N., Klein M.A., Lauder A., Bujdoso R. (2004). Accelerated Prion Disease in the Absence of Interleukin-10. J. Virol..

[57] Hasebe R., Suzuki A., Yamasaki T., Horiuchi M. (2014). Temporary upregulation of anti-inflammatory cytokine IL-13 expression in the brains of CD14 deficient mice in the early stage of prion infection. Biochem. Biophys. Res. Commun..

[58] Fernández-Botrán R., Ahmed Z., Crespo F.A., Gatenbee C., Gonzalez J., Dickson D.W., Litvan I. (2011). Cytokine expression and microglial activation in progressive supranuclear palsy. Park. Relat. Disord..

[59] Ponomarev E.D., Maresz K., Tan Y., Dittel B.N. (2007). CNS-Derived Interleukin-4 Is Essential for the Regulation of Autoimmune Inflammation and Induces a State of Alternative Activation in Microglial Cells. J. Neurosci..

[60] Fenn A.M., Hall J.C., Gensel J.C., Popovich P.G., Godbout J.P. (2014). IL-4 Signaling Drives a Unique Arginase+/IL-1 + Microglia Phenotype and Recruits Macrophages to the Inflammatory CNS: Consequences of Age-Related Deficits in IL-4R after Traumatic Spinal Cord Injury. J. Neurosci..

